# A Novel Approach to Develop Lager Yeast with Higher NADH Availability to Improve the Flavor Stability of Industrial Beer

**DOI:** 10.3390/foods10123057

**Published:** 2021-12-08

**Authors:** Xin Xu, Chengtuo Niu, Chunfeng Liu, Jinjing Wang, Feiyun Zheng, Qi Li

**Affiliations:** 1 Key Laboratory of Industrial Biotechnology, Ministry of Education, School of Biotechnology, Jiangnan University, Wuxi 214122, China; xxin@jiangnan.edu.cn (X.X.); Chengtuoniu@jiangnan.edu.cn (C.N.); Chunfengliu@jiangnan.edu.cn (C.L.); Jinjingwang@jiangnan.edu.cn (J.W.); Feiyunzheng@jiangnan.edu.cn (F.Z.); 2 Laboratory of Brewing Science and Engineering, Jiangnan University, Wuxi 214000, China

**Keywords:** beer, flavor stability, lager yeast, ARTP, DNP

## Abstract

Flavor stability is important for beer quality and extensive efforts have been undertaken to improve this. In our previous work, we proved a concept whereby metabolic engineering lager yeast with increased cellular nicotinamide adenine dinucleotide hydride (NADH) availability could enhance the flavor stability of beer. However, the method for breeding non-genetically modified strains with higher NADH levels remains unsolved. In the current study, we reported a novel approach to develop such strains based on atmospheric and room temperature plasma (ARTP) mutagenesis coupled with 2,4-dinitrophenol (DNP) selection. As a result, we obtained a serial of strains with higher NADH levels as well as improved flavor stability. For screening an optimal strain with industrial application potential, we examined the other fermentation characteristics of the mutants and ultimately obtained the optimal strain, YDR-63. The overall fermentation performance of the strain YDR-63 in pilot-scale fermentation was similar to that of the parental strain YJ-002, but the acetaldehyde production was decreased by 53.7% and the resistance staling value of beer was improved by 99.8%. The forced beer aging assay further demonstrated that the favor stability was indeed improved as the contents of 5-hydroxymethylfurfural in YDR-63 was less than that in YJ-002 and the sensory notes of staling was weaker in YDR-63. We also employed this novel approach to another industrial strain, M14, and succeeded in improving its flavor stability. All the findings demonstrated the efficiency and versatility of this new approach in developing strains with improved flavor stability for the beer industry.

## 1. Introduction

Flavor is the main quality characteristic for beer and requires the flavor-active components presented as raw materials or developed by yeast metabolism to be controlled within a certain range in order to maintain flavor balance [1,2,3]. For many breweries, this issue can be addressed through good manufacturing practices. However, during the shelf life of beer, numerous reactions take place, resulting in a decrease of fresh flavor notes and the appearance of typical aged flavors [4,5,6]. Hence, improving the flavor stability of beer during its shelf life is of great concern for brewers as it is important for a commercial beer to have a consistent sensory experience and satisfy the expectations of consumers at all times.

Aldehydes such as (*E*)-2-nonenal, 5-hydroxymethylfurfural, hexanal, and acetaldehyde are characterized as the aged flavor components in beer, and higher concentrations of these aldehydes greatly impair flavor stability [7,8,9]. Thus, many strategies have been applied to reduce the production of these aldehydes during industrial manufacturing, for example, reducing the aeration levels of wort, limiting the time of boiling and cooling, and increasing the inoculation volume of cells [7]. Notably, the natural reducing activity of yeast can also facilitate the reduction of these components [10]. Hence, breeding lager yeast with higher reducing activity is of great interest. In our previous work, we proposed that higher reducing activity could be directly replaced by the higher reducing nicotinamide adenine dinucleotide hydride (NADH) level in yeast, and confirmed that increasing cellular NADH availability in lager yeast could reduce the production of numerous aldehydes, thereby enhancing the flavor stability of beer [11,12]. However, due to the relevant laws and regulations in the food industry, these genetically modified strains cannot be applied in industrial beer production. Hence, breeding non-genetically modified strains with higher NADH availability presents a new challenge.

There are many powerful methods to create microbial mutations, of which ARTP mutagenesis is the new developed technology with the advantages of high efficiency, consistency, nontoxicity, environmental-friendliness, and low cost [13,14]. Therefore, generating non-genetically modified mutant yeast cells with this method would be a good choice. In addition, prior findings suggested that the uncoupler 2,4-dinitrophenol (DNP) could block NADH oxidation and affect cell growth [15,16]; therefore, it might be a promising selective marker for NADH perturbation varients.

In this study, we first explored the effectiveness of DNP on selecting ARTP mutants with NADH perturbation. Then we performed a typical strain screening procedure to obtain the optimal strain with improved flavor stability and industrial potential. Next, we conducted a pilot-scale fermentation with the mutant and parental strain, and compared the fermentation performance and sensory difference between these two strains. Furthermore, we carried out a forced aging assay to prove the shelf-life of the beer produced by the mutant strain was prolonged. Finally, we employed this new approach for breeding other industrial strains with improved flavor stability.

## 2. Materials and Methods

### 2.1. Strains and Chemicals

In this study, two industrial lager yeasts were used. *Saccharomyces pastorianus* strain YJ-002 was provided by Yanjing Brewery Group Co., Ltd. (Beijing, China), while *S. pastorianus* strain M14 was from TSINGTAO Brewery Group Co., Ltd. (Qingdao, China). The other strains were derived from these two strains by ARTP mutagenesis. All strains were cultivated in Yeast extract Peptone Dextrose (YPD) media at 28 °C for cell growth or fermented in 14 °P wort at 11 °C. The chemicals and standards used in this study were purchased from Sigma-Aldrich (Shanghai, China) or Sinopharm (Shanghai, China).

### 2.2. ARTP Mutagenesis

The ARTP system was purchased from Siqingyuan Bio-technology Co., Ltd. (Beijing, China), consisting of a radio frequency power supplier, a co-axial type plasma generator, a gas supplier, and a sample plate. The default parameters were set as the following: the flow rate of helium was 15.0 L/min, the power input was 100 W, and the distance between the plasma torch nozzle and sample plate was 2 mm. To explore the optimal treatment time, 10 µL cells (OD_600_ was adjusted to 1.0) were pipetted on metal plates and submitted to ARTP for 0, 15, 30, 45, 60, and 75 s, respectively. Then, the metal plates were washed with 1 mL YPD media and diluted appropriately. Next, 100 μL dilutions were spread on YPD agar plate. After 3–5 days cultivation, the survival colonies were counted to calculate the death rate. The optimal treatment time was chosen when the death rate reached 80% to 90%. To explore the lethal concentration of DNP, 100 μL cells (OD_600_ was adjusted to 1.0) were spread on YPD agar plate containing different concentration of DNP. The result indicated the minimum inhibitory concentrations of DNP in the media for YJ-002 and M14 were 0.10 and 0.15 mM, respectively. Based on the above results, the cells were submitted to ARTP with the optimal treatment time, and spread on a YPD agar plate supplied with a lethal concentration of DNP, and cultivated at 28 °C for 5–7 days.

### 2.3. Beer Fermentation

The lab-scale fermentation process was performed in a 250 mL Erlenmeyer flask filled with 150 mL wort. The wort was prepared as previously described and the final wort was adjusted to 14 °P [12]. The pitching rate was around 1.5 × 10^7^ cells/mL, and the fermentation temperature was set at 11 °C. Samples were collected at the end of main fermentation (day 7) for analysis. During the primary selection process, the fermentation for each strain was performed once only, but when the selection process entered into the fermentation stability test, three experiments for each strain were carried out.

The Pilot scale fermentation process was performed in cylindroconical fermentation vessels (CCTs). The wort volume in each CCT was 50 L, whereas the gross CCT capacity was 65 L. Worts were produced using basic pilsner malts from COFCO Corporation (JiangYin, China). The mashing process took place at a standard scale temperature, i.e., 50 °C for 40 min, 65 °C for 60 min, 72 °C for 20 min, and 78 °C for 10 min. Afterwards, the mash was transferred to a lauter tun for boiling. During the boiling process, hops were added at 10 min and 60 min after the boiling began. The wort was cooled by a plate type heat exchanger to 11 °C and aerated with compressed sterile air under 1 bar of pressure pushed through the wort line for 20 min. The final gravity of the wort in each CCTs was about 14.2 °P. Primary fermentation was performed at 11 °C without top pressure, and when the apparent extract had decreased to 3.5 °P, the CCTs was locked to keep the pressure at 0.1 Mpa. The reduction of diacetyl to the concentration of 0.10 mg/L marked the end of fermentation and the beginning of maturation process. The maturation process was performed in the same tank and the temperature was set at 1 °C. Three samples were taken with a one-day interval for analysis.

### 2.4. Analytical Methods

To determine the cellular NADH/NAD^+^ ratio, the cells were collected after two days cultivation and centrifuged at 3000× *g* for 10 min at 4 °C, and washed twice with pre-chilled distilled water. Determination of the NADH/NAD^+^ ratio was carried out using the commercial cofactor test kit (No. NAD-2-Y; Comin, Suzhou, China), following the instructions of the manufacturer. Each sample was tested three times.

The thiobarbituric acid (TBA) method was used to evaluate the degree of beer staling and performed as previously described [17]. Degassed fermentation liquid of 2 mL was added to 2 mL of thiobarbutiric reactive reagent containing 0.33% (*w*/*v*) TBA in 50% (*v*/*v*) acetic acid. The mixture was incubated in a 60 °C water bath for 60 min. The absorbance of the solution was measured at 530 nm, with 2 mL of 50% (*v*/*v*) acetic acid as the blank. The flavor freshness period was assessed using the resistance staling value (RSV): RSV = 1/4(12/ΔTBA_12_ + 24/ΔTBA_24_ + 36/ΔTBA_36_ + 48/ΔTBA_48_) [18]. △TBA_t_ means the sample was kept in 60 °C water bath for *t* h before the TBA measurement.

Ethanol production and Real degree of attenuation (RDF) were determined by an Anton Paar Alcohol meter equipped with Alcolyzer Beer ME and DMA 4500 M (Graz, Austria). Diacetyl content was determined by distillation according to the National Standard of the Peoples Republic of China (GB/T 4928-2008). Fusel alcohols, esters, and acetaldehyde were detected using headspace gas chromatography (GC-2010 PerkinElmer TurboMatrix 16, Shimadzu, China) with 3-heptanone as an internal standard [19].

A forced aging assay was performed to evaluate the tendency of beer aging during shelf-life [20]. Sample were prepared by incubating the beer at 37 °C for 10 days, and 5-Hydroxymethylfurfural (5-HMF) was measured by high-performance liquid chromatography using the column ZORBAX Eclipse XDB-C18 4.6 × 250 mm (Agilent, Shanghai, China).

Sensory analysis was performed using a comparison test, with the beer sample produced by YDR-63 compared to the beer produced by YJ-002. Profile tests involved the evaluation of attributes of the beer, including aroma (esters, alcohols, malts, and hops), tastes (sweetness, acidity, bitterness, and acerbity), and mouthfeel (body, fullness, balance, and freshness). Each item was scored from 0 to 9. The sensory analysis panel consisted of six employees from Yanjing Brewery whose standard job was to routinely assess the sensory quality of beer. The analyzed samples were taken at the end of storage and at the end of the 10-day forced aging treatment.

## 3. Results

### 3.1. DNP Serves as a Selective Marker for the NADH Perturbation Variants

**Figure 1:**
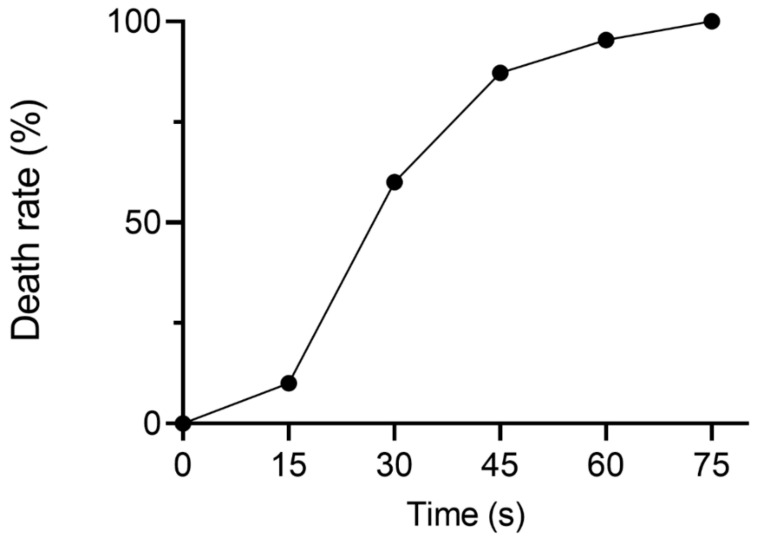
The death rate of YJ-002 at different times of ARTP treatment.

In this study, ARTP mutagenesis was used to create mutant libraries. To explore the optimal ARTP treatment time for YJ-002, the death rates of YJ-002 at different treatment times were assessed. The death rate of YJ-002 reached 87.2% after treatment for 45 s, but reached 95.4% when treated for 60 s (Figure 1). Thus, 45 s was chosen as the optimal treatment time and used in subsequent ARTP mutagenesis studies.

**Figure 2:**
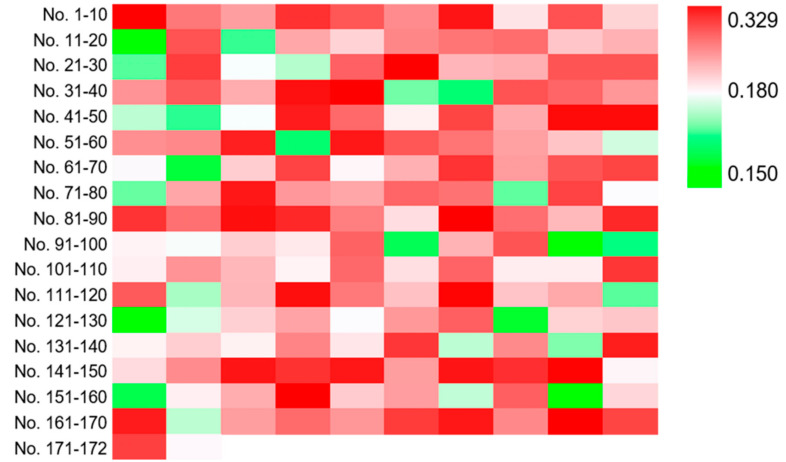
Heatmap for the NADH/NAD^+^ ratios of the mutants selected from the DNP plate. The NADH/NAD^+^ ratio of YJ-002 was 0.180. The strains with an increased NADH level are marked in red, and those with a decreased value are marked in green.

Then, three runs of ARTP mutagenesis were performed on strain YJ-002 and 172 colonies were obtained from the YPD agar plate containing 0.10 mM DNP. To investigate whether DNP could serve as a selective marker for NADH perturbation variants, the mutants were cultured in YPD media for 2 days and the cells were collected to measure the cellular NADH/NAD^+^ ratio. Of these 172 mutants, 142 strains exhibited higher NADH/NAD^+^ ratios than the parental strain YJ-002 with the values from 0.180 to 0.329, while 30 mutants exhibited lower NADH/NAD^+^ ratios with the values from 0.150 to 0.180 (Figure 2). This result suggested that DNP could effectively serve as a selective marker for NADH perturbation variants, especially for mutants with higher NADH levels, with the positive rate approximately at 82.6% based on our results.

### 3.2. Screening the Optimal Strain with Industrial Potential

**Figure 3:**
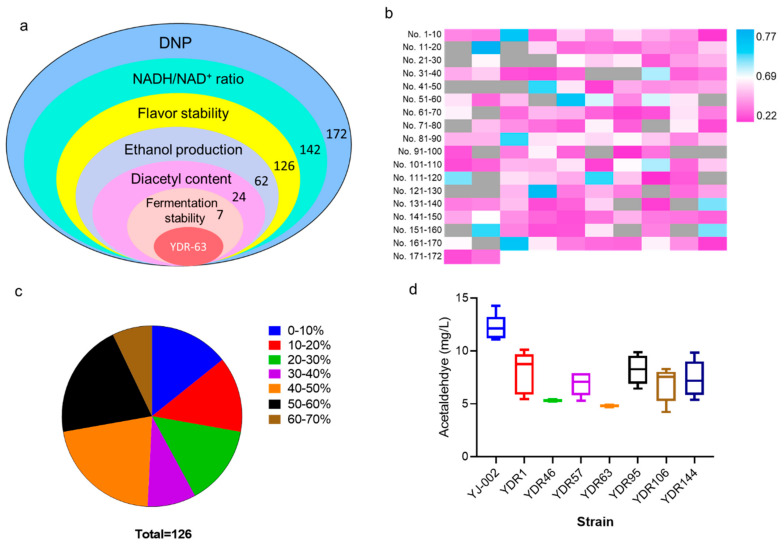
Screening an optimal strain with improved flavor stability. (**a**) Schematic representation of the entire selection. (**b**) Heat map for the TAB value of the mutants; strains with reduced TBA value than YJ-002 are marked in pink, strains with increased TBA value are marked in blue, strains with reduced NADH levels are marked in grey, and the TBA values have not been detected. (**c**) The reduction rates of the TBA value in those strains with decreased TBA value (126 of 142) were calculated to reflect the improvement in the flavor stability. (**d**) Fermentation stability is an important criterion for the industrial producing strain; therefore, five runs of fermentation were carried out and the fluctuations in acetaldehyde production were compared to judge this characteristic.

To screen an optimal strain with improved flavor stability as well as industrial potential, several indices were considered (Figure 3a). Lab-scale fermentation was performed using the 142 mutants with an increased NADH/NAD^+^ ratio, and the TBA method was first employed to assess the flavor stability of the fermentation liquid. A total of 126 strains exhibited improved flavor stability (Figure 3b; Sheet S1). Of these 126 strains, 108 strains showed great improvements in flavor stability, with the reduction rates of TBA values exceeding 10% (Figure 3c). To continue the screening process, 62 strains with TBA reduction rates of over 40% were chosen for next selection (Figure 3a). Ethanol is the main product obtained from yeast during alcoholic fermentation, and lower ethanol production generally indicates incomplete carbohydrate utilization resulting in greater economic losses to breweries [21,22]. By measuring the ethanol content of these 62 strains, 38 strains with reduced ethanol production exceeding 5% were removed from the candidate pools (Figure 3a, Sheet S1). Diacetyl is formed from the spontaneous oxidative decarboxylation of α-acetolactate and higher concentration contributes negatively to the beer with a buttery flavor [2,23]. We tested the diacetyl content in the fermentation liquid, and 17 strains with increased diacetyl production were eliminated (Sheet S1). Consequently, seven strains remained after this selection run.

For industrial scale-producing strains, the fermentation stability was important as the yeast cells were collected and reused for fermentation for more than four runs. Acetaldehyde was the most abundant aldehyde in beer and was identified as a key contributor to beer staling [11]. Therefore, the fermentation stability of these seven mutants was evaluated by analyzing fluctuations in acetaldehyde production over five runs of fermentation. The acetaldehyde concentration of these seven strains was lower than that of YJ-002 during five runs of continuous fermentation (Figure 3d). However, large fluctuations in acetaldehyde production were identified in the different generations of strains YDR-1, YDR-57, YDR-95, YDR-106, and YDR-144. In contrast, acetaldehyde content was almost constant among the different generations of YDR-46 and YDR-63. Strain YDR-63 was chosen as the optimal strain for this round of screening, as the production of acetaldehyde in YDR-63 within each generation was lower than that inYDR-46.

### 3.3. Pilot Scale Fermentation

**Figure 4:**
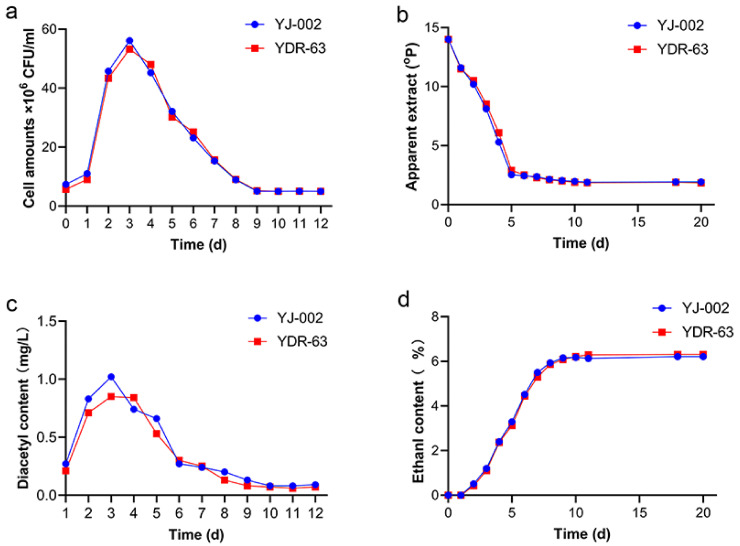
Fermentation performance of the optimal strain YDR-63 and parental strain YJ-002. (**a**) Cell amounts; (**b**) apparent extract; (**c**) diacetyl content; (**d**) ethanol production. Three samples were collected at each time point, and the data are shown as mean ± SD.

**Figure 5:**
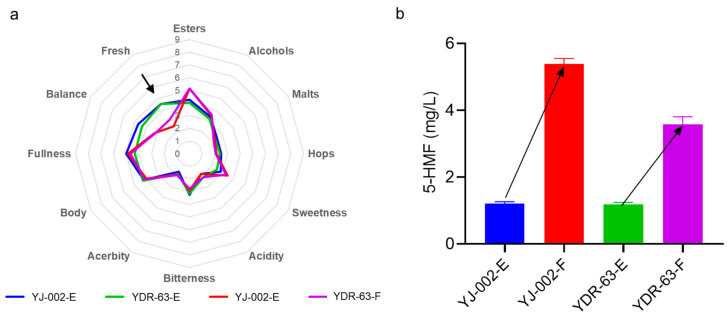
(**a**) Sensory analysis of the beer produced by the strain YDR-63 and strain YJ-002. (**b**) Detecting the levels of 5-HMF in beer samples to reflect the beer aging degree. Samples collected at the end of storage were marked with the tail E while the samples with forced aging treatment were marked with the tail F.

To understand the actual fermentation performance of the mutant strain YDR-63, we conducted pilot-scale fermentation in CCTs. YDR-63 shared a similar growth and fermentation ability to the parental strain YJ-002 (Figure 4a,b,d). Nonetheless, YDR-63 had a lower peak diacetyl content, and the fermentation process ended one day earlier than in the case of YJ-002 (Figure 4c). After storage, several parameters related to beer quality were measured. The RDF of YDR-63 was approximately 69.29%, which was slightly higher than that of YJ-002 (68.73%) (Table 1). The ethanol production of these two strains was consistent with that of RDF. Alcohol production and ethyl acetate production decreased slightly in the YDR-63. As a causal effect, there was little sensory difference between the beer samples produced by YJ-002 and YDR-63 (Figure 5a). Notably, the acetaldehyde production in YDR-63 was reduced by 53.69%, and the RSV value increased from 50.23 to 100.37%, indicating the beer produced by YDR-63 could have a prolonged shelf-life.

To prove this, we performed a forced aging assay with these beer samples. After 10 days treatment, we found that levels of the aging indicator 5-HMF in YJ-002 samples was 1.57-fold than that of the 5-HMF levels in YDR-63 samples (Figure 5b), strongly indicating that the staling degree of beer caused by YJ-002 was much higher than that caused by YDR-63. Concordantly, the freshness of the YJ-002 produced beer was greatly decreased with a typical staling note akin to carboard. In contrast, this note in YDR-63 samples was not prevalent (Figure 5a). Overall, these results indicated that the flavor stability of beer produced by YDR-63 was remarkably improved during storage.

### 3.4. Application of DNP Selection to Other Industrial Strain

The new approach was adopted for another industrial strain, M14, to further demonstrate its feasibility for breeding strains with improved flavor stability. A similar procedure was used and led to obtaining the optimal strain MDR-17 (Sheet S2). The fermentation parameters for this strain in pilot-scale fermentation are shown in Table 1, and the findings demonstrated that flavor stability was greatly improved.

## 4. Discussion

Beer staling is of great concern to brewers as it leads to an irreversible change in flavor. Therefore, to improve the flavor stability of beer during shelf life, it is imperative to reduce the production of aldehydes in the final product. Acetaldehyde production was the main target in this study, as it accounts for approximately 90% of the carbonyl compounds in beer and has a clear metabolic pathway. In previous studies, strains with lower acetaldehyde production and improved flavor stability have been developed, based on ARTP mutagenesis coupled with 4-methylpyrazole (inhibitor of alcohol dehydrogenase 2) selection or disulfiram (inhibitor of aldehyde dehydrogenase) selection [18,24,25]. However, other aldehydes, such as (*E*)-2-nonena and 5-Hydroxymethylfurfura, have also been identified as key factors contributing toward beer staling, despite their extremely low concentrations [9,26,27]. Thus, reducing the production of these aldehydes is also of great interest to the beer production industry. In the current study, through metabolic engineering of the lager yeast, we found that increasing the cellular NADH levels could address this issue because the reductases for these aldehydes were all NADH-dependent [28]. Hence, developing a new approach to screen strains with higher NADH levels by establishing an effective selective marker has important implications.

DNP is already used as a dye, as well as in wood preserver, herbicides, munitions, and photographic developer, and was also initially popularized as a weight loss drug, as the consumption of DNP led to significant weight loss [15]. The mechanism underlying this weight loss property was that it increases the basal metabolic rate by uncoupling oxidative phosphorylation and stimulating the glycolysis rate [29,30]. However, this chemical was soon labeled “not fit for human consumption” owing to many adverse effects [31]. In cellular metabolism, both the oxidation phosphorylation and glycolysis are involved in NADH accumulation; thus, we assumed that this chemical could be used as a selective marker for strains with cellular NADH perturbation. We were able to prove this hypothesis and found that the majority of these strains had increased cellular NADH levels. This result not only suggests a novel use of DNP, but also provided us with an opportunity to develop a new approach for screening the strains with improved flavor stability. The feasibility of this new approach for breeding strains with improved flavor stability was further demonstrated using two industrial strains from different breweries, and the obtained results were found to be validated.

Overall, this study provided a new and practical approach for screening strains with higher NADH levels, and two industrial strains with greatly improved flavor stability were obtained. We expect that this new approach will be useful in developing more such industrial strains in the future, thereby reducing economic losses to breweries and increasing consumers’ satisfaction.

## Figures and Tables

**Table 1:** Parameters after the fermentation in CCTs with 14 °P wort (mean ± SD, *n* = 3, * *p* < 0.05).

	YJ-002	YDR63	M14	MDR17
Real attenuation (%)	68.73 ± 0.12	69.29 ± 0.08 *	65.49 ± 0.19	65.04 ± 0.05 *
Diacetyl (mg/L)	0.08 ± 0.01	0.07 ± 0.01	0.05 ± 0.01	0.06 ± 0.01
Ethanol (%)	6.21 ± 0.16	6.31 ± 0.09	5.56 ± 0.15	5.52 ± 0.03
Acetaldehyde (mg/L)	10.56 ± 0.04	4.89 ± 0.03 *	14.16 ± 0.03	5.14 ± 0.05 *
Ethyl acetate (mg/L)	18.42 ± 0.11	17.33 ± 0.17 *	13.24 ± 0.08	13.17 ± 0.05
Isoamyl acetate (mg/L)	1.27 ± 0.03	1.59 ± 0.05 *	0.35 ± 0.04	0.33 ± 0.05
*n*-Propanol (mg/L)	13.82 ± 0.21	12.78 ± 0.23 *	8.23 ± 0.25	8.57 ± 0.18
Isoamyl alcohol (mg/L)	54.55 ± 0.23	50.35 ± 0.20 *	40.62 ± 0.26	41.91 ± 0.37 *
Isobutanol (mg/L)	9.17 ± 0.18	6.98 ± 0.13 *	6.43 ± 0.05	6.71 ± 0.08 *
RSV	50.23	100.37 *	61.55	133.24 *

## Supplementary Materials

The following are available online at https://www.mdpi.com/article/10.3390/foods10123057/s1, Table S1.1: NADH -NAD; Table S1.2: TBA Value; Table S1.3: Ethanl Content (%); Table S1.4: Diacetyl Content (mg·L-1); Table S2.1: NADH-NAD; Table S2.2; TBA Value; Table S2.3; Ethanol (%); Table S2.4; Diaceyl; Table S2.5: Acetaldehyde.
