# Supplementary material for: A Novel Approach to Develop Lager Yeast with Higher NADH Availability to Improve the Flavor Stability of Industrial Beer
Source: Foods. 2021 Dec 8;10(12):3057. doi: 10.3390/foods10123057 (PMC8701400; doi:10.3390/foods10123057)
Supplement: Supplementary file 1 [file foods-10-03057-s001.zip › foods-1412421-supplementary.pdf]

Supplementary Materials

Table S1.1. NADH -NAD.

No.										
1-10	0.32	0.259	0.236	0.299	0.278	0.247	0.313	0.194	0.281	0.204
11-20	0.154	0.28	0.168	0.231	0.204	0.251	0.261	0.265	0.213	0.22
21-30	0.169	0.292	0.179	0.174	0.272	0.329	0.223	0.227	0.279	0.278
31-40	0.242	0.276	0.228	0.315	0.327	0.17	0.165	0.28	0.27	0.242
41-50	0.175	0.168	0.179	0.31	0.26	0.187	0.288	0.23	0.319	0.319
51-60	0.24	0.249	0.308	0.164	0.312	0.277	0.26	0.235	0.214	0.176
61-70	0.181	0.16	0.208	0.289	0.183	0.226	0.298	0.238	0.279	0.288
71-80	0.169	0.232	0.312	0.24	0.232	0.27	0.262	0.169	0.289	0.18
81-90	0.297	0.264	0.316	0.303	0.255	0.199	0.32	0.264	0.221	0.303
91-100	0.185	0.179	0.208	0.192	0.271	0.162	0.225	0.27	0.156	0.166
101-110	0.188	0.242	0.221	0.185	0.267	0.198	0.271	0.189	0.189	0.296
111-120	0.276	0.173	0.222	0.316	0.258	0.215	0.322	0.213	0.23	0.169
121-130	0.15	0.177	0.206	0.233	0.181	0.239	0.273	0.159	0.205	0.213
131-140	0.185	0.208	0.187	0.251	0.193	0.296	0.175	0.248	0.17	0.309
141-150	0.2	0.247	0.313	0.298	0.313	0.237	0.313	0.3	0.323	0.184
151-160	0.1619	0.187	0.228	0.327	0.21	0.237	0.175	0.273	0.157	0.202
161-170	0.309	0.174	0.237	0.266	0.241	0.293	0.312	0.249	0.327	0.288
171-172	0.29	0.183								
YJ-002	0.18									

Table S1.2. TBA Value.

No.										
1-10	0.41	0.39	0.75	0.33	0.58	0.42	0.61	0.49	0.43	0.24
11-20		0.77		0.59	0.35	0.37	0.35	0.43	0.41	0.57
21-30		0.67			0.68	0.57	0.64	0.31	0.46	0.51
31-40	0.49	0.57	0.29	0.27	0.33			0.71	0.33	0.38
41-50				0.73	0.63	0.26	0.49	0.43	0.45	0.48
51-60	0.62	0.34	0.53		0.75	0.70	0.41	0.70	0.63	
61-70	0.66		0.35	0.52	0.49	0.33	0.26	0.29	0.62	0.39
71-80		0.54	0.43	0.36	0.31	0.66	0.32		0.65	0.28
81-90	0.51	0.51	0.73	0.62	0.65	0.60	0.67	0.56	0.41	0.56
91-100	0.30		0.39	0.66	0.33		0.23	0.36		
101-110	0.28	0.33	0.52	0.51	0.62	0.26	0.68	0.71	0.34	0.56
111-120	0.72		0.61	0.56	0.49	0.73	0.55	0.22	0.31	
121-130			0.52	0.76	0.38	0.51	0.39		0.35	0.47
131-140	0.39	0.41	0.54	0.28	0.33	0.58		0.25		0.72
141-150	0.48	0.69	0.43	0.34	0.29	0.36	0.51	0.38	0.37	0.33
151-160		0.73	0.40	0.29	0.30	0.63		0.40		0.72
161-170	0.68		0.75	0.64	0.39	0.35	0.34	0.66	0.43	
171-172	0.28	0.36								
YJ-002	0.690									

Table S1.3. Ethanl Content (%).

No.										
1-10	5.1	4.33		4.56						5.11

11-20					4.71	4.51	4.88		5.09		
21-30								5.23			
31-40			4.62	4.12	4.33				4.88	4.87	
41-50						5.34					
51-60		5.1					5.2				
61-70			5.22			4.77	4.65	4.32		4.89	
71-80										4.77	
81-90									4.95		
91-100	4.85		4.75		4.92		4.52	4.77			
101-110	4.83	4.95				4.99			4.73		
111-120								4.85	4.85		
121-130					4.93		4.25		4.97		4.47
131-140	5.12	5.17		4.77	4.86			4.98			
141-150				5.3	4.32	4.77		4.63	4.95	5.1	
151-160			5.19	4.99	4.53						
161-170					4.91	4.35	5.11			4.72	
171-172	3.27	4.18									
YJ-002	5.13										

Table S1.4. Diacetyl Content (mg·L-1).

**No.**										
1-10	0.18									0.23
11-20							0.22		0.24	
21-30								0.3		
31-40									0.25	
41-50						0.17				
51-60		0.24					0.15			
61-70			0.19							0.23
71-80										
81-90									0.26	
91-100					0.17					
101-110		0.23				0.18				
111-120										
121-130					0.22				0.21	
131-140	0.23	0.23						0.25		
141-150				0.2					0.22	0.23
151-160			0.25	0.24						
161-170					0.23		0.21			
171-172										
YJ-002	0.21									

Table S2.1. NADH-NAD.

**No.**										
1-10	0.251	0.233	0.273	0.384	0.337	0.153	0.244	0.173	0.486	0.356
11-20	0.133	0.293	0.533	0.175	0.227	0.267	0.428	0.328	0.182	0.193
21-30	0.165	0.366	0.377	0.264	0.215	0.343	0.432	0.223	0.244	0.161
31-40	0.263	0.122	0.393	0.402	0.232	0.265	0.514	0.226	0.243	0.249
M14	0.241									

Table S2.2. TBA Value.

No.										
1-10	0.49		0.54	0.37	0.4		0.48		0.33	0.36
11-20		0.59	0.28			0.35	0.29	0.37		
21-30		0.55	0.32	0.34		0.41	0.39		0.61	
31-40	0.33		0.62	0.33		0.55	0.58		0.54	0.34
M14	0.57									

Table S2.3. Ethanol (%).

No.								
1-10			4.49				5.12	4.91
11-20		4.85		4.35	4.69	4.78		
21-30		4.87	4.58		4.77			
31-40	4.22		4.92					3.78
M14	4.83							

Table S2.4. Diaceyl.

No.							
1-10						0.11	0.13
11-20		0.12		0.11	0.1		
21-30		0.11	0.13	0.12			
31-40			0.11				
M14	0.13						

Table S2.5. Acetaldehyde.

No.							
1-10						6.27	7.95
11-20		10.77		5.17	7.73		
21-30		9.85	10.12	6.31			
31-40			12.85				
M14	12.16						
